# Effects of vitamin D supplementation on ovulation and pregnancy in women with polycystic ovary syndrome: a systematic review and meta-analysis

**DOI:** 10.3389/fendo.2023.1148556

**Published:** 2023-08-01

**Authors:** Meina Yang, Xiaoyang Shen, Danhua Lu, Jin Peng, Siyu Zhou, Liangzhi Xu, Jing Zhang

**Affiliations:** ^1^ Department of Obstetrics and Gynecology, West China Second University Hospital, Sichuan University, Chengdu, China; ^2^ Key Laboratory of Birth Defects and Related Diseases of Women and Children (Sichuan University), Ministry of Education, Chengdu, China; ^3^ Reproductive Endocrinology and Regulation Laboratory, West China Second University Hospital, Sichuan University, Chengdu, China; ^4^ The Joint Laboratory for Reproductive Medicine of Sichuan University, The Chinese University of Hong Kong, Chengdu, China; ^5^ National Clinical Research Center for Geriatrics, Department of Gerontology and Geriatrics, West China Hospital, Sichuan University, Chengdu, China

**Keywords:** vitamin D, polycystic ovary syndrome, pregnancy, ovulation, meta-analysis

## Abstract

**Objective:**

To evaluate the effect of vitamin D supplementation on pregnancy and ovulation in patients with polycystic ovary syndrome.

**Method:**

We searched Pubmed, Medline (via Ovid, 1974 to 2020), EMBASE (via Ovid, 1974 to 2020), Cochrane Central Register of Controlled Trials (via Ovid), Web of Science, CNKI, WangFang and the Vip database from inception until April 2021. Two researchers independently screened articles, collected data and evaluated the quality, with Review manager 5.3 for meta-analysis.

**Results:**

Totally 20 randomized controlled studies with 1961 subjects were included. Meta analysis showed that pregnancy rate [RR=1.44 (1.28, 1.62), p<0.00,001], ovulation rate [RR=1.42 (1.14, 1.78), p=0.002] and matured oocytes rate [RR=1.08 (1.03, 1.13), p=0.002] of vitamin D supplementation group were significantly higher than those of control group. Meanwhile, early miscarriage rate [RR=0.44 (0.30, 0.66), p<0.00,001], androgen level [MD=-2.31 (-3.51, -1.11), p=0.0002], luteinizing hormone [MD=-1.47 (-2.57, -0.36), p=0.009], follicle stimulating hormone [MD=-0.15 (-0.24, -0.05), p=0.002], and premature delivery rate [RR=0.38, 95% CI (0.21, 0.70), p=0.002] were declined significantly than the controls. However, only one article suggested that the progesterone [MD=6.52 (4.52, 8.52), p<0.05] in the vitamin D intervention group was increased. There was no notable difference in the biochemical pregnancy rate [RR=0.95 (0.55, 1.63), p=0.84], gestational hypertension rate [RR=0.40, 95% CI (0.15, 1.11), p=0.08], gestational diabetes mellitus rate [RR=0.27, 95% CI (0.05, 1.39), p=0.11], fertilization rate [RR=1.05 (1.00, 1.10), p=0.04], cleavage rate [RR=1.03 (0.99, 1.06), p=0.17], high-quality embryo rate [RR=1.08 (0.98, 1.20), p=0.10], endometrial thickness [MD=0.10], 77 (-0.23, 1.77), p=0.13], estrogen level [MD=-0.34 (-1.55, 0.87), p=0.59], LH/FSH [MD=-0.14, 95% CI (-0.48, 0.20), p=1.00] and anti-Mullerian hormone [MD=-0.22 (-0.65, 0.21), p=0.32].

**Conclusion:**

Vitamin D supplementation contribute to the higher pregnancy and ovulation rates, and lower androgen, LH, FSH and early miscarriage rates in women with PCOS, regardless of the use of ovulation induction drugs or assisted reproductive technologies. However, no significant improvement was observed in fertilization rate or cleavage rate. Due to the limitation in quality of involved studies, more high-quality RCTs are needed for further validation.

**Systematic review registration:**

https://www.crd.york.ac.uk/PROSPERO, identifier CRD42021250284.

## 1 Introduction

Polycystic ovary syndrome (PCOS) is the most common endocrine disorder in women of childbearing period, and 75% of these patients suffer from infertility (1). Existing treatments of infertility in PCOS mainly include monitoring ovulation, ovulation induction, ovarian perforation and assisted reproduction technologies (2). In addition, the supplementation of some nutrients has received some attention as an adjunction, such as vitamins, unsaturated fatty acids, minerals, etc. (3). Notably, about 67 – 85% of PCOS patients have concomitant vitamin D deficiency (4). What’s more, vitamin D deficiency may be involved in the pathogenesis of insulin resistance and metabolic syndrome in PCOS (5). These days, vitamin D supplementation draw some attentions in the preparation for pregnancy in PCOS patients, and the possible mechanism relies on its protection against oxidative stress and keep calcium ion levels to maintain the cell’s resting potential, which in turn benefits fertilization, cleavage, implantation, and placental formation (6).

However, current conclusions regarding the effect of vitamin D supplementation on pregnancy rates are inconsistent. Some studies have shown that vitamin D supplementation was able to increase pregnancy rates in PCOS patients, while others have proved that vitamin D supplementation had no significant improvement (7). Based on this argument, the present study systematically collated the existing evidence of vitamin D adjuvant treatment for infertility in PCOS patients, with the view of seeking evidence for whether vitamin D improves the pregnancy situation of PCOS patients and providing a basis for clinical medication.

## 2 Methods

### 2.1 Eligibility criteria, study selection and data extraction

This article followed the agreement registered in the International Prospective Systems Review Register (No. CRD42021250284).

#### 2.1.1 Criteria included

##### 2.1.1.1 Study type

Randomized controlled study.

##### 2.1.1.2 Subjects

Patients with PCOS who had fertility requirements. The diagnostic criteria of PCOS follows the Rotterdam criteria in 2003, that at least two of the following three criteria were met:

###### 2.1.1.2.1 Ovulatory dysfunction (oligoovulation/anovulation);

####### 2.1.1.2.1.1 Hyperandrogenism serum testosterone level ≥ 2.5 nmol/l or clinical manifestations of hyperandrogenism (hirsutism: modified Ferriman Gallwey score ≥ 8, Acne or alopecia);

####### 2.1.1.2.1.2 Polycystic ovary like changes (defined as ≥ 12 follicles (2 – 9 mm)) in one or two ovaries and/or ovarian volume>10 cm^3^ under ultrasound. Other causes of hyperandrogenism (8) (such as congenital adrenal hyperplasia, Cushing’s syndrome, androgen secreting tumors, etc.) or ovulation disorders (for example: functional hypothalamic amenorrhea, thyroid disease, hyperprolactinemia, premature ovarian insufficiency, etc.) were excluded.

##### 2.1.1.3 Interventions

Placebo, metformin, short acting contraceptives, ovulation induction drugs and assisted reproductive technology were defined as control group. Vitamin D supplemented based on control group (dosage form, time and dosage are not limited) were considered as the intervention group.

##### 2.1.1.4 Outcome indicators

Main outcomes were clinical pregnancy rate and ovulation rate, and secondary outcomes were related outcomes like cumulative pregnancy rate, biochemical pregnancy rate, pregnancy complications, term delivery rate, premature delivery rate, matured oocytes rate, sex hormone level and adverse events. It was suggested that the ovulation manifestations include progesterone level ≥ 19 nmol/l,or after the dominant follicle (≥ 1.2mm) under ultrasound, the dominant follicle disappeared, collapsed or shrank by ≥ 50%, and a small amount of fluid accumulated in the rectal fossa. If the basal body temperature rose for 3 days and no ovulation was observed by ultrasonography, luteinized unruptured follicle syndrome (LUFS) would be diagnosed. The follow-up duration was 6 months.

#### 2.1.2 Excluding criteria

Conference summary, animal experiment, cohort study, retrospective study and non- randomized controlled intervention study were excluded.

#### 2.1.3 Data extraction

Two researchers (Yang Meina, Peng Jin) independently evaluated the articles and collected information. Inconsistencies were judged through discussion with the third author (Zhang Jing). The two researchers independently collected the following data: basic characteristics (author, year, country); Characteristics of subjects (age, number of people in each group) Outcome indicators (pregnancy related indicators: pregnancy rate, biochemical pregnancy rate, early miscarriage rate, incidence of pregnancy complications, assisted reproduction related indicators: fertilization rate, cleavage rate, high-quality embryo rate, fetal premature delivery rate, ovulation related indicators: ovulation rate, matured oocytes rate, endometrial thickness, sex hormone level and adverse events). We sent emails to study authors for more information.

### 2.2 Search strategy

We searched Pubmed, MEDLINE(via Ovid, 1974 to 2020), EMBASE (via Ovid, 1974 to 2020), Cochrane Central Register of Controlled Trials (CENTRAL) (via Ovid), Web of Science, CNKI, WangFang and the Vip database from inception until April 2021. We applied “vitamin d” “vitamin d1” “vitamin d2” “vitamin d3” “1, 25(OH)2 vitamin D” “cholecalciferol” “Polycystic Ovary Syndrome” “Randomized Controlled Trial” as search terms. The search strategy is available in **Supplementary Information**.

### 2.3 Data synthesis and analysis

Data analyses were conducted with the software Review Manager 5.3. For dichotomous data (ovulation and pregnancy rate for example), we used relative risk (RR). When it comes to continuous data (luteinizing hormone (LH) and follicle-stimulating hormone (FSH) and so on), mean difference (MD) was applied. When different scales were taken for the same, we performed standardized mean difference (SMD) instead. MD, if not mentioned, was calculated from the standard error, interquartile range or 95% confidence interval. If there were at least 5 studies, we synthesized the data. Cochrane risk of bias tool was implemented to access the quality of the evidence. We applied I² test to examine the heterogeneity. The fixed-effects model was applied when I² <50%, otherwise the random-effects model was conducted. Sensitivity analysis was utilized to distinguish the source of heterogeneity in Stata 12.0. We extracted data from images with Engauge Digitizer 4.1 if necessary. Begg’s and Egger’s test were performed to access the public bias.

## 3 Results

### 3.1 Studies selection and the flow chart

**Figure 1:**
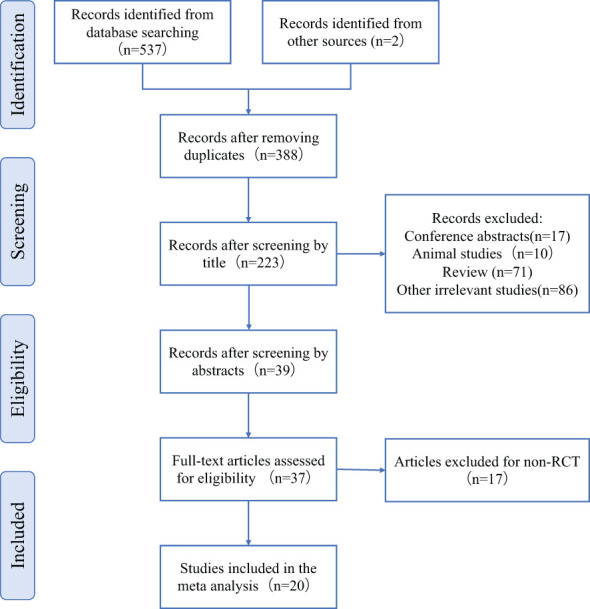
Flowchart of study selection.

A total of 557 articles were retrieved, including 555 from the database and 2 from the references. After screening by EndnoteX8, 388 articles were left after removing duplicate articles (n=169).39 articles were left after removing conference abstract (n=17), animal experiment (n=10), and irrelevant articles (n=86) after browsing the title. We read the abstracts and carefully screened out non RCT studies (n=17) and no full text studies (n=2). A total of 20 articles were included:8 in English and 12 in Chinese (**Figure 1**).

### 3.2 Characteristics of included studies

**Table 1** presents the basic characteristics of the 20 RCTs including 1961 subjects. The average age of control group was 28.83 ± 4.35 years old, and it was 28.61 ± 4.56 years of age in the vitamin D intervention group. There was no statistical difference in the age of the subjects in studies. The baseline level of vitamin D in the control group was 15.40 ± 6.50 on average, and that in the intervention group was 14.22 ± 4.86. Both groups were in a state of vitamin D deficiency, which were comparable. Only two studies mentioned whether the subjects were secondary infertility, which was balanced and comparable between groups. The other articles did not mention the number of previous pregnancies. All included studies did not mention whether subjects experienced any pregnancy complication in the past. The follow-up time was from 2 weeks to 3 months. Two of the studies reported the delivery situation and subjects with fetal heart rate under ultrasound 5 weeks after embryo implantation and followed up until delivery. The intervention of vitamin D were intramuscular injection or long-term oral administration. The average daily dose varies from 200 IU to 10000 IU, and the intervention duration varies from a single dose, 8 weeks, 12 weeks and 24 weeks. In terms of outcome indicators, 18 studies reported pregnancy rate, 9 included ovulation rate, and 7 investigated matured oocytes rate (**Table 1**).

**Table 1:** Characteristics of the included studies.

Study		N	Population		Follow-up	Basic treatments		Experimental			Control	Outcome analyzed
Author, year	Country		Mean age (y)	BMI				Length	Daily dose (vitamin D3)	Form		
Bonakdaran, S. 2012 (9)	Iran	51	25.2±7.9	25.3±5.1	3m	None	Detecting Ovulation	12wks	0.5 ug calcitriol/d	Oral tablets	Placebo	ovulation outcome, T, DHEAS, 25(OH)D, body mass, BP, FBS, INS, HOMA-IR, Ca, P, PTH, OGTT
Rashidi, B. 2009 (8)	Iran	60	25.8±4.6	25.2±3.8	3m	metformin 150mg/d		12wks	Calcium 1000 mg/d, vitamin D 400 IU/d	Orally	None	pregnancy rates, regularity of menses, number of large follicles (>= 14 mm)
Firouzabadi, Rd 2012 (10)	Iran	100	28.4±4.2	26.9±2.1	6m	metformin 1500 mg/d		24wks	Calcium 1000 mg/day and Vitamin D 100000 IU/month	Oral drops	None	pregnancy, menstrual regularity, follicle diameter, BMI, serum 25-OH-vitamin D level
Li Yaguang 2017 (11)	China	118	32.7±4.5	NM	NM	metformin 150mg/d		4wks	1200 IU/d	Orally	None	total ovulation rate, endometrial thicknes, endometrial blood flow rate
Wang Xiaohong 2015 (12)	China	108	32.4±9.8	NM	NM	metformin 150mg/d		4wks	1200 IU/d	Oral drops	None	ovulation cycle number, ovulation occurring and total ovulation rate, endometrial thicknes, endometrial blood flow rate
Liu Shengxian 2020 (13)	China	122	30.5±5.2	NM	NM	Bushen Yanggong decoction		12wks	800 IU/d	Orally	None	the ovulation rate and pregnancy rate, endometrial thicknes, P, E, SOD level
Liu Hui 2020 (14)	China	68	29.5±2.9	23.6±1.3	6m	COC (Drospirenone and Ethinylestradiol Tablets)	COC	12wks	6000 IU/week	Orally	None	pregnancy rate, vitamin D level, FSH, LH, T
Wei Chenghou 2021 (15)	China	72	27.9±6.9	NM	2w	COC (Ethinylestradiol and Cyproterone Acetate Tablets), metformin 1500mg/d		12wks	200 IU/d	Oral drops	None	the ovulation rate and pregnancy rate, LH, FSH, T, AMH, menstrual regularity
Li Yan 2019 (16)	China	70	29.5±2.9	23.6±1.3	NM	COC (Ethinylestradiol and Cyproterone Acetate Tablets), metformin 150mg/d		12wks	2000 IU/d	Orally	None	the ovulation rate and pregnancy rate, menstrual regularity, LH, FSH, E2
Wang Li 2018 (17)	China	120	29.1±5.4	24.2±2.7	2m	Letrozole, HMG	Ovulation-inducing drugs	12wks	2000 IU/d	Oral drops	None	the ovulation rate and pregnancy rate, LH, FSH, endometrial thicknes, FPG, FINS, HOMA-IR, TC, TG, LDL-C, HDL-C, SDS, SAS
Zhang Miaomiao 2015 (18)	China	60	NM	23.0±4.0	NM	Letrozole, COC (Ethinylestradiol and Cyproterone Acetate Tablets)		12wks	1200 IU/d	Orally	None	pregnancy rate, LH, FSH, E2, AMH, endometrial thicknes, FPG, FINS, HOMA-IR
Abdulameeryahya, A. 2019 (7)	Iraq	32	24.71±4.07	27.59±4.63	1m	Clomiphene citrate, HCG		8wks	10000IU/d	Oral tablets	CO-enzyme Q10 200mg	the ovulation rate and pregnancy rate, LH, FSH, T, AMH
Rasheedy, R. 2020 (19)	Egypt	186	29.8±3.5	27.9±1.3	3m	clomiphene citrate 100 mg/d, norethisterone, calcium 2500 mg/d		16wks	10,000 IU twice weekly	Orally	Placebo	the ovulation rate and pregnancy rate, Biochemical pregnancy, Clinical pregnancy, Clinical pregnancy losses
Chen Ruili 2018 (20)	China	141	23.1±3.2	24.6±2.8	2w	COC, HCG, Letrozole		12wks	1200IU/d	Oral drops	None	the ovulation rate and pregnancy rate, endometrial thicknes, early abortion rate, FSH, LH, T, E2, AMH, FPG, FINS, HOMA-IR, 25(OH)D
Bhatti, Z. I. 2019 (21)	Pakistan	82	28.95±4.4	NM	NM	Frozen thawed embryo transfer.	ART	once	200000IU/once	NM	None	the ovulation rate and pregnancy rate,endometrial thicknes, Embryo quality
Fan Furong 2020 (22)	China	110	27.9±5.1	NM	till delivery	Gonadotrophin-releasing hormone (GnRH) agonist +hCG		8wks	1200IU/d	Oral drops	None	the pregnancy rate, the average number of ovum obtained, the ovum maturation rate, the fertilization rate, the cleavage rate, the high-quality embryo rate, the early abortion rate, the incidence of hydramnios and premature rupture of membranes
Liu Shengxian 2018 (23)	China	128	27.1±3.8	24.8±1.2	till delivery	COH		8wks	1200 IU/d	Oral drops	None	Pregnancy, Retrieved oocyte, Matured oocytes %, Fertilization rate, High quality embryo %, the early abortion rate, premuture birth rate, the incidence of pregnancy-induced hypertension, the incidence of gestational diabetes mellitus, the length of new born, fetal weight
Liu Shengxian 2020 (13)	China	120	27.6±4.1	25.0±1.3	NM	COH long protocol		8wks	800 IU/d	Oral drops	None	Pregnancy, Retrieved oocyte, Matured oocytes %, Fertilization rate, High quality embryo %, the early abortion rate, premuture birth rate, the incidence of pregnancy-induced hypertension, the incidence of gestational diabetes mellitus, the length of new born, fetal weight
Fatemi, Farnaz 2017 (24)	Iran	105	28.1±3.7	26.13±2.99	2w	(GnRH) agonist -the “long” protocol: combined oral contraceptive pill (COCP), ICSI		8wks	3300 IU/d	Oral tablets	Placebo	Pregnancy, clinical pregnancy and implantation rate, Retrieved oocyte, Matured oocytes % , Retrieved embryo, High quality embryo %, Fertilization rate %, vitamin D, MDA and TAC levels
Asadi, M. 2014 (25)	Iran	110	26.29±3.8	NM	3m	IUI–COH, clomiphene citrate, HMG, HCG		once	300000 IU /once	Intragluteal injection	Placebo	the pregnancy rate, The endometrial thickness, number of dominant follicles, duration of IUI cycles, E

NM, not mentioned; COC, combination oral contraceptive; ART, Assisted reproduction technologies; IUI, intra uterine insemination; COH, controlled ovarian hyperstimulation; HCG, human chorionic gonadotropin; HMG, human menopausal gonadotropins; BP, blood pressure; SOD, superoxidase dismutase; FSH, follicle-stimulating hormone; LH, luteinizing hormone; T, testosterone; P, progesterone; E, estradiol; AMH, anti-Müllerian hormone; FPG, fasting plasma glucose; FINS, fasting insulin; OGTT, oral glucose tolerance test; SDS, the scores of the self- rating depression scale; SAS, self- rating anxiety scale; MDA, malondialdehyde; TAC, total antioxidant capacity (TAC).

**Figure 2:**
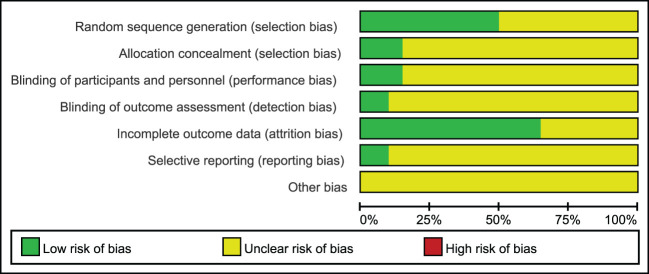
Risk of bias graph: review authors' judgements about each risk of bias item presented as percentages across all included studies.

**Figure 3:**
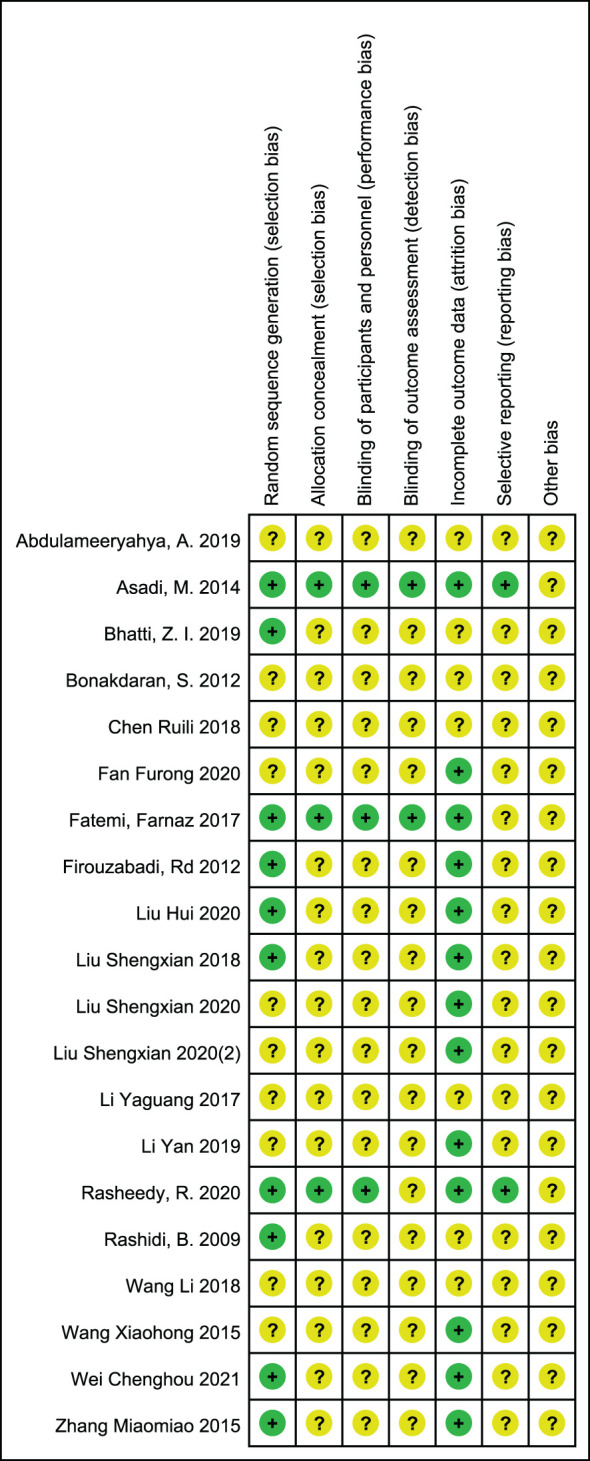
Risk of bias summary: review authors' judgements about each risk of bias item for each included study.

The included studies were summarized in **Figures 2**, **3** according to Cochrane risk of bias tool. Random methods used in 8 studies were random number table, 2 studies used blocked randomization, 1 study was conducted by blocked randomization, and the remaining 9 studies only mentioned randomization in words. In terms of allocation concealment, 2 studies used opaque envelopes, 2 articles mentioned it in words, and the remaining 16 did not mention it. 4 articles were blinding of participants, and the rest 16 articles did not mention, only one article used the blinding of personnel, and the other 19 articles failed to mention. None of the 20 articles mentioned the bias of selective reporting of research results and other sources bias.

### 3.3 Meta analysis

#### 3.3.1 Pregnancy related outcomes

##### 3.3.1.1 Pregnancy rate

**Figure 4:**
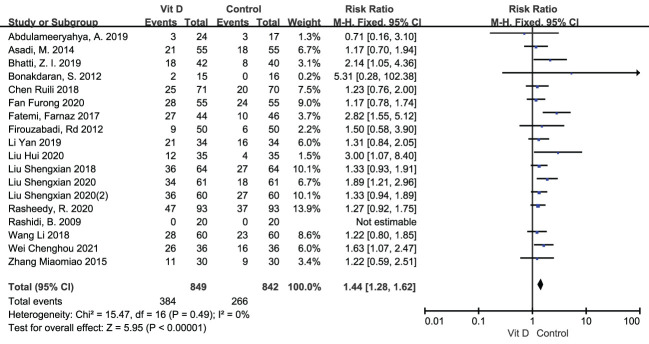
Forest plot of the pregnancy rate.

The clinical pregnancy rate was reported in 18 RCTs, including 849 participants in the intervention group and 842 cases in the control group. Meta analysis result of fixed effect model showed that after vitamin D supplementation, the pregnancy rate of PCOS patients significantly increased [RR=1.44, 95% CI (1.28, 1.62), p<0.00001] comparing with the control group (**Figure 4**; **Table 2**).

**Table 2:** Vitamin D supplement compared with control for PCOS patients.

Outcome		Study included number	Heterogeneity		Effect model	Meta analysis	
			P value	I^2^		Relative effect (95%CI)	P value
Pregnancy related outcomes	Pregnancy rate	18	0.49	0%	Fixed	1.44 (1.28, 1.62)	<0.00001
Ovulation related outcomes	Ovulation rate	9	0.01	59%	Random	1.42 (1.14, 1.78)	0.002
Sex hormone related outcomes	Testosterone	7	<0.00001	97%	Random	-2.31 (-3.51, -1.11)	0.0002
	Estradiol	5	<0.00001	97%	Random	-0.34 (-1.55, 0.87)	0.59
	LH	8	<0.00001	93%	Random	-1.99 (-2.25, -1.73)	<0.00001
	FSH	7	0.71	0%	Fixed	-0.15 (-0.24, -0.05)	0.002
	LH/FSH	7	0.41	0%	Fixed	-0.14 (-0.48, 0.20)	1.00
	AMH	4	0.02	68%	Random	-0.22 (-0.65, 0.21)	0.32

IVF, in vitro fertilization; LH, luteinizing hormone; FSH, follicle-stimulating hormone; AMH, Anti-mullerian hormone.

The following conclusions were drawn from the subgroup analyses of patient characteristics (baseline vitamin D level, testosterone level, and history of infertility) and vitamin D supplementation characteristics (average daily dose, calcium was supplemented at the same time, duration and frequency of medication) (see **Table 3**):

**Table 3:** Subgroup analyses of pregnancy rate.

Subgroups	Number of study	RR (95%CI)	I^2^ (%)
Infertility			
Yes	13	1.88 [1.48, 2.38]	7
Not mentioned	5	1.89 [1.24, 2.87]	16
Baseline vitamin D			
≥20ng/ml(50nmol/l)	1	1.22 (0.59, 2.51)	Not applicable
<20ng/ml(50nmol/l)	10	1.57 (1.29, 1.90)	21
Not mentioned	7	1.36 (1.16, 1.58)	0
Baseline testosterone			
Hyperandrogenemia	5	1.52 (1.16, 1.98)	0
Normal	4	1.39 (1.14, 1.69)	0
Daily dose			
<2000 IU/d	7	1.43 (1.22, 1.68)	0
≥ 2000 IU/d	8	1.43 (1.19, 1.71)	22
Period of treatment			
Once	2	1.47 (0.98, 2.22)	47
4 weeks	0	Not estimable	Not applicable
8 weeks	5	1.42 (1.17, 1.74)	44
12 weeks	9	1.49 (1.24, 1.81)	0
>12 weeks	2	1.30 (0.96, 1.77)	0
Timing of treatment			
Daily	12	1.36 (1.18, 1.56)	0
Intermittently	6	1.62 (1.30, 2.02)	45
Intervention			
Vitamin D	16	1.44 (1.27, 1.62)	3
Calcium with vitamin D	2	1.50 (0.58, 3.90)	0

RR, relative risk.

We divided patients into 2 subgroups based on whether they had infertility or not. As a result, a total of 13 studies involved patients with infertility, and the vitamin D intervention group had an increased pregnancy rate when compared with the control group [RR = 1.88, 95% CI (1.48, 2.38), P < 0.00001, I²= 7%]. While those studies without mention infertility in PCOS patients showed the same trend [RR = 1.89, 95% CI (1.24, 2.87), P = 0.003, I² = 16%].

According to *Method for vitamin D deficiency screening* published by National Health Commission of the People’s Republic of China in June 2020, serum vitamin D content ≥ 20 ng/ml (50 nmol/L) was considered as the normal level whileunders12 ng/ml (30 nmol/L) was defined as vitamin D deficiency status, and between as vitamin D insufficiency status. Only 1 study reported PCOS patients with normal vitamin D at baseline, and there was no significant difference of the pregnancy rate between the vitamin D intervention group and the control group [RR = 1.22, 95% CI (0.59, 2.51), P = 0.59]. Patients in ten studies were in vitamin D insufficiency condition at baseline, and pregnancy rates improved significantly in PCOS patients compared with controls after vitamin D intervention [RR = 1.57, 95% CI (1.29, 1.90), P < 0.00001, I² = 21%]. Whereas 7 studies did not report baseline of vitamin D levels, the pregnancy rate improved in the intervention group compared with the control group [RR = 1.36, 95% CI (1.16, 1.58), P = 0.0001, I² = 0%].

We applied subgroup analysis according to subjects with or without hyperandrogenism at baseline, the result revealed pregnancy rates consistency in patients with [RR = 1.52, 95% CI (1.16, 1.98), P = 0.002, I² = 0%] or without [RR = 1.39, 95% CI (1.14, 1.69), P = 0.0009, I² = 0%] hyperandrogenism.

Subgroup analysis performed by whether daily dose above 2000IUor not. Results showed no difference about pregnancy rates between the lower daily dose (<2000IU/d) [RR=1.43, 95%CI (1.22, 1.68),p<0.00001, I²=0%] and the higher daily dose(>2000IU/d) [RR=1.43, 95%CI (1.19, 1.71),p=0.0001, I²=22%].

Subgroup analysis according to the period of treatment unveiled that 8 weeks [RR = 1.42, 95% CI (1.17, 1.74), P = 0.0004, I² = 44%] or 12 weeks [RR = 1.49, 95% CI (1.24, 1.81), P < 0.00001, I² = 0%] of vitamin D supplementation both improved pregnancy rates. However, either single dose [RR = 1.47, 95% CI (0.98, 2.22), p = 0.06, I² = 47%] or more than 12 weeks [RR = 1.30, 95% CI (0.96, 1.77), p=0.09, I²=0%] did not ameliorate the pregnancy rate. On the other aspect, both continuous daily administration [RR = 1.36, 95% CI (1.18, 1.56), P < 0.0001, I² = 0%] and intermittent administration [RR = 1.62, 95% CI (1.30, 2.02), P < 0.0001, I² = 45%] of vitamin D elevated pregnancy rates in PCOS.

Vitamin D supplementation alone [RR = 1.44, 95% CI (1.27, 1.62), P < 0.00001, I² = 3%] did rise the pregnancy rate. However, it was not significantly changed by concomitant calcium supplementation [RR = 1.50, 95% CI (0.58, 3.90), P = 0.41, I² = 0%], probably due to the small number of relative studies.

##### 3.3.1.2 Cumulative pregnancy rates

Cumulative pregnancy rate equals the number of pregnancies (per patient, clinical pregnancy, or after transfer of all embryos from one oocyte retrieval cycle)/number of all oocyte retrieval cycles × 100% (26). Only one study reported the cumulative pregnancy rate (19). After the first cycle of induced ovulation, both groups had the same cumulative pregnancy rate (0%). Cumulative pregnancy rates were 13% and 12% in the vitamin D intervention and control groups after the second cycle, respectively. After the third cycle, it was 29% in the intervention group and 24% in the control group. We drew data and discovered no statistical difference between both groups for the three cycles, specifically for the first cycle [RR = 0.99, 95% CI (0.89, 1.09)], the second cycle [RR = 0.96, 95% CI (0.83, 1.09)], and the third cycle [RR = 0.87, 95% CI (0.71, 1.07)] (**Table 2**).

#### 3.3.2 Ovulation related outcomes

##### 3.3.2.1 Ovulation rate

**Figure 5:**
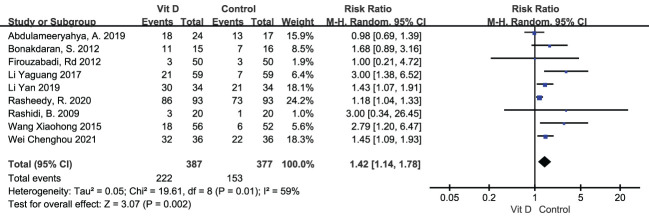
Forest plot of the ovulation rate.

Ovulation rate was displayed in 9 studies, including 387 subjects in the vitamin D group and 377 in the control group. Random effects model showed that ovulation rate was apparently improved with vitamin D supplementation [RR=1.42, 95%CI (1.14, 1.78), p=0.002, I²=59%] (**Table 2**; **Figure 5**).

##### 3.3.2.2 Sex hormone related outcomes

###### 3.3.2.2.1 Luteinizing hormone

A total of 8 studies revealed luteinizing hormone levels, including 345 subjects in the intervention group and 340 in the control group. The random effects model showed that LH levels were significantly lower in individuals of the vitamin D supplemented group[MD= -1.47, 95%CI (-2.57, -0.36), p=0.009, I²=93%]. We carried out the sensitivity analysis for obvious heterogeneity. After removing the result in (13), I² decreased to 0%, while LH was still lower in the intervention group [MD= -1.31, 95%CI (-1.60, -1.01), p<0.00001, I²=0%] (**Table 2**).

###### 3.3.2.2.2 Follicle-stimulating hormone

Follicle stimulating hormone levels were mentioned in 7 studies, involving 284 in the intervention group and 280 in the control group. A fixed effects model resulted that FSH levels were noticeable lower in the vitamin D supplemented group [MD=-0.15, 95%CI (-0.24, -0.05), p=0.002, I²=0%] (**Table 2**).

###### 3.3.2.2.3 LH/FSH

Seven studies showed both LH and FSH levels, consisted of 284 patients in the vitamin D supplementation group and 279 patients in the control group. LH/FSH was calculated and the fixed effects model found that LH/FSH was not significantly different between two groups [MD=-0.14, 95%CI (-0.48, 0.20), p=1.00, I²=0%] (**Table 2**).

###### 3.3.2.2.4 Anti-mullerian hormone

There were 4 studies reported anti-mullerian hormone, including 155 participants in the intervention group and 150 in the control group. The random effects model showed that AMH levels were not significantly changed in the vitamin D supplemented group compared with the control group [MD=-0.22, 95%CI (-0.65, 0.21), p=0.32, I²=68%] (**Table 2**).

###### 3.3.2.2.5 Testosterone

Testosterone levels were reported in 7 studies. A random effect model displayed a significant decrease in the vitamin D supplementation group compared with the control group [MD=-2.31, 95%CI(-3.51, -1.11), p=0.0002, I²=97%]. Heterogeneity remained large after subgroup analyses according to the test period: with basal testosterone levels under a random effects model [MD=-1.48, 95%CI (-2.94, -0.01), p=0.05, I²=95%] and the test period not mentioned [MD=-3.40, 95%CI (-5.99, -0.80), p=0.01, I²=98%], respectively (**Table 2**).

###### 3.3.2.2.6 Estradiol

Estradiol levels were assessed in 5 literatures, involving 251 patients supplemented with vitamin D and 250 patients in the control group. Random effects model resulted that estradiol levels were not significantly different in two groups [MD=-0.34,95%CI (-1.55, 0.87), p=0.59, I²=97%]. Subgroups analysis according to the examining period demonstrated the same trend under random effects model meta-analysis. There was no noticeable difference between the two groups about basal estradiol levels [MD=0.32, 95%CI (-0.07, 0.71), p=0.11, I²=66%] and estradiol levels that the test time not mentioned [MD=-1.34, 95%CI (-5.49, 2.81), p=0.53, I²=99%] (**Table 2**).

In summary, we predominantly found vitamin D supplementation could ameliorate outcomes like the premature delivery rate and the matured oocytes rate. While other indicators did not alter significantly (the early miscarriage rate, biochemical pregnancy rate, the fertilization rate, the incidence of gestational hypertension and gestational diabetes mellitus (GDM), the cleavage rate, the high quality embryo rate and endometrial thickness) (**Supplementary Information**).

#### 3.3.3 Adverse events

Only 4 studies reported the adverse events. One trail showed no obvious adverse reactions in both intervention and control groups. Two of them demonstrated ovarian hyperstimulation syndrome (OHSS) occurred in both groups during ovulation induction, but there was no significant difference between the vitamin D intervention group and the control group. One research revealed that the control group had allergic reactions to short-acting oral contraceptives.

In general, no trail had severe adverse event for vitamin D per se.

### 3.4 Bias

**Figure 6:**
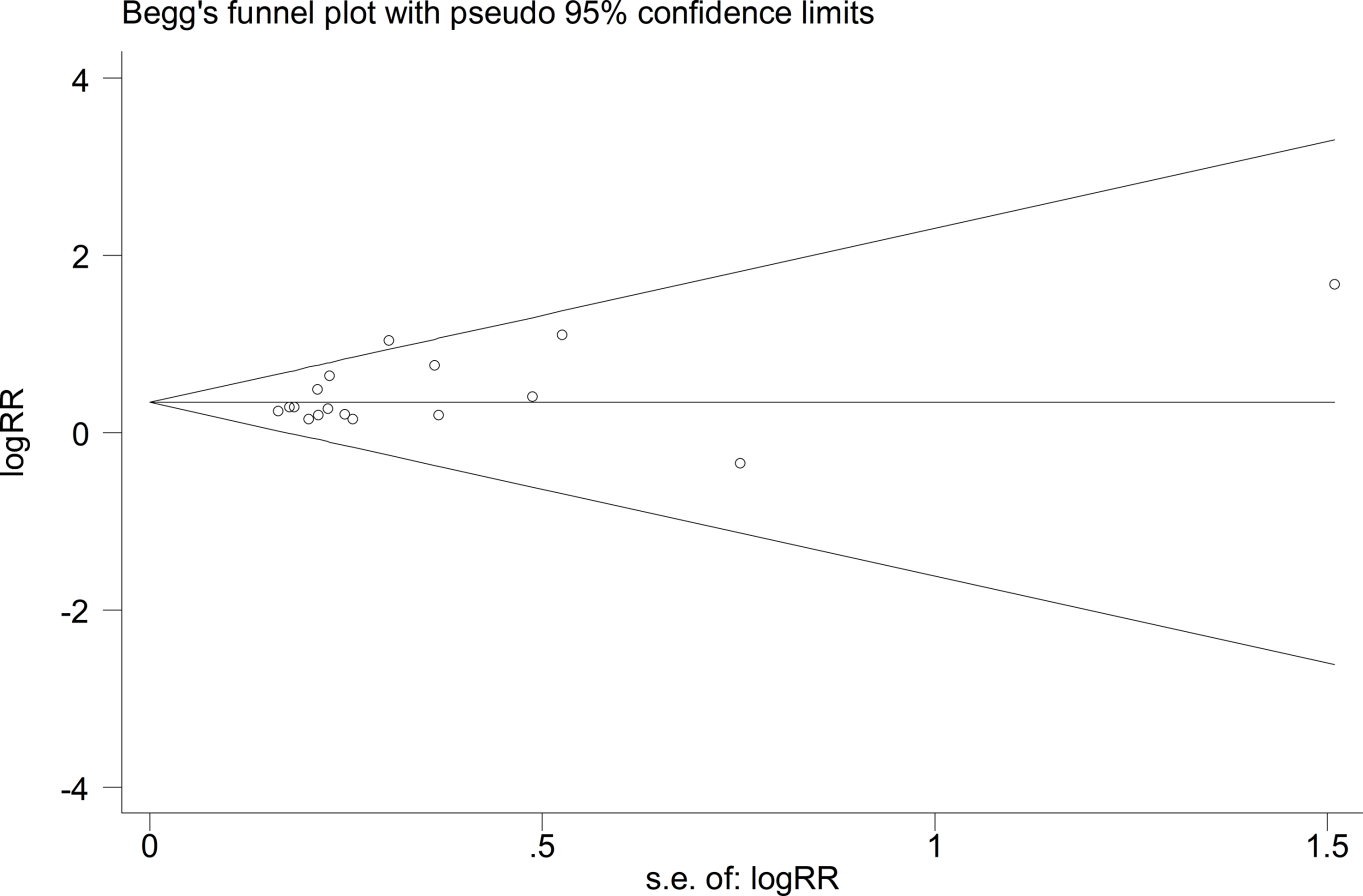
Begg’s test.

**Figure 7:**
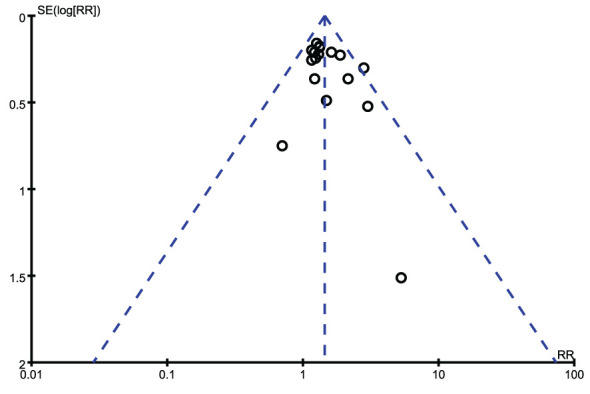
Funnel plots.

We exerted funnel plots, Begg’s tests and Egger’s tests. No public bias was found in our research (**Figures 6**, **7**).

## 4 Discussion

PCOS patients are generally experiencing vitamin D deficiency, and it is not known whether vitamin supplementation is beneficial for pregnancy in PCOS patients till now. This is the first systematic review and meta-analysis of vitamin D supplementation on pregnancy in PCOS patients. A total of 20 RCTs were included. There were 1961 subjects in total, no statistical difference was discovered between the intervention group and the control group in terms of age and vitamin D level at baseline. This systematic review, based on the existing RCTs, provides evidence for PCOS patients to take vitamin D supplements or not before pregnancy.

The results of meta-analysis found that the pregnancy rate, ovulation rate and matured oocytes rate of vitamin D supplementation group were significantly higher than those of the control group. The premature delivery rate, testosterone, LH and FSH decreased evidently. Only one trial suggested that the level of progesterone in vitamin D supplementation group increased. There was no remarkable difference with vitamin D supplemented in chemical purity rate, fertilization rate, cleavage rate, high-quality embryo rate, endometrial thickness, serum estradiol and AMH.

### 4.1 Pregnancy related outcomes

In this study, the pregnancy rate of PCOS patients was apparently increased with vitamin D supplementation, but vitamin D had no significant impact on other indicators related to assisted reproductive technology, such as fertilization rate, cleavage rate and high-quality embryo rate. To best of our knowledge, there is no systematic review on the gestation of PCOS patients with vitamin D supplementation. Existing results are dominantly on the correlation between vitamin D levels and pregnancy rate in IVF, and the conclusions are not consistent. Specifically, vitamin D levels in serum and follicular fluid may improve, reduce or have no effect on pregnancy rate (5), and the reason for the inconsistency may be the difference of vitamin D levels in patients themselves. The mechanism of how vitamin D improve the pregnancy rate of PCOS patients is not clearly demonstrated. In combination with other results of this study, we speculated that supplementation of vitamin D may improve the pregnancy rate of PCOS patients by mitigating inflammation in granulosa cells, ameliorating ovulation and increasing endometrial thickness.

This study also indicated that the incidence of gestational hypertension and gestational diabetes mellitus (GDB) in the vitamin D supplementation group was not significantly different from that in the control group, but the premature delivery rate was reduced. However, only three articles included in this study mentioned pregnancy complications. Thus the results should be treated with caution. The existing meta-analysis found that vitamin D supplementation during pregnancy can reduce the risk of preeclampsia, but no difference was found about the incidence of gestational diabetes mellitus (27). The different findings between this study and the previous study may because of populations (PCOS or healthy gravidas), timing of vitamin D supplementation (before or during maternity) and doses they used. Some researchers have pointed out that vitamin D plays a role in reducing blood pressure and glucose level. A meta-analysis found that the diastolic blood pressure of adolescents decreased after vitamin D supplement (28). The potential mechanism is that vitamin D reduces the production of endothelium-derived contractile factor and ultimately decreases blood pressure by up-regulating endothelial nitric oxide synthase (eNOS) activity, reducing the levels of reactive oxygen species (ROS) and cyclooxygenase-1 (COX-1) (29). In addition, vitamin D deficiency is positively related to the risk of GBD (30). The underlying mechanism of vitamin D’s improvement on glucose metabolism is (1) to stimulate the expression of insulin receptor, thus increasing the insulin sensitivity of liver, muscle and adipose tissue (2) to stimulate the nuclear vitamin D receptor, thus raising the production of GLUT-4 (3) to promote calcium-dependent insulin secretion (4) to inhibit the production of cytokines from macrophages, and protect islet cells from damage.

Moreover, we found that vitamin D supplementation may reduce the incidence of premature delivery in PCOS offspring in present study. However, it is noteworthy that only two studies have reported on this outcome and were completed by the same researcher, which may cause bias. At present, there is no other research reported the effect of vitamin D supplementation on the occurrence of premature delivery of the fetus in PCOS women, but a meta-analysis found that the risk of premature delivery of pregnant women with vitamin D deficiency is increased (31). Although more studies are still needed to verify, there are some possible grounds for the reduction of premature delivery rate of fetus by vitamin D supplementation for gravidas. Research demonstrates that vitamin D deficiency of pregnant women is positively correlated with the increased risk of premature delivery (32, 33), and vitamin D may reduce the incidence of premature delivery *via* NF- κ B, which plays an anti-inflammatory role and reduces infections (34).

### 4.2 Ovulation rate

We found that vitamin D supplementation improved ovulation rate in PCOS patients. Berry et al. claims that 70% infertile PCOS women have a lower level of vitamin D than the healthy (35). Besides, studies unveil that vitamin D deficiency is positively associated with the number of mature oocytes (36) and the ovulation rates (35, 37, 38). Nevertheless, different concepts still exist. Evidence states serum vitamin D in PCOS women are similar to controls (39). The discrepancy may originate from diverse races and the specific type of PCOS. Except for vitamin D receptor and sex hormone related mechanism that will be mentioned later, the mechanism of VD affecting ovulation is that VD may down regulate AMH receptor II, thus promote the activation and maturation of oocytes (40).

### 4.3 Sex hormone related outcomes

In addition to the above gestation related indicators, the effect of vitamin D on the level of sex hormones in patients with PCOS also deserves attention. In this study, compared with the control group, testosterone, LH and FSH in vitamin D supplementation group decreased significantly, while progesterone level increased, but estradiol, AMH and endometrial thickness did not change.

We found that the serum testosterone level decreased in vitamin D intervention group. Studies manifests that in women with PCOS, the level of 25-hydroxyvitamin D (25 (OH) D) is negatively correlated with testosterone (29). A meta-analysis involving 183 PCOS patients suggests serum testosterone decreases after vitamin D supplementation (41), which complies with this study. Similarly, Nahla et al. demonstrates 50,000 IU per week of VD can decrease testosterone in overweight PCOS women (42). The mechanisms related to vitamin D and testosterone are not obvious. Possible mechanisms might be the improvement of insulin resistance, thus interrupting the vicious cycle of insulin resistance-hyperandrogenism. The increase of aromatase activity promote the conversion of androgen to estrogen, which is described later. In addition, the etiology of PCOS is linked to the effect of vitamin D receptor (VDR) gene polymorphism on LH, SHBG concentration, testosterone level, insulin resistance and serum insulin level (41). In terms of the molecular mechanism, vitamin D deficiency increases the activity of the PI3K/Akt pathway in ovarian tissue, thus interfering with the selection of dominant follicles. What’s more, hyperandrogenism lead to increasing AR activity. Decreased ARA70 (androgen receptor-associated protein 70, which is necessary for androgen receptor (AR) and VDR transcription activity) subsequently inactivates free testosterone by promoting the transcription of VDR/RXR (retinoid X receptor) complex, which induces a vicious cycle of hyperandrogenism. Vitamin D supplementation breaks this cycle by up-regulating VDR (43).

There was no remarkable difference in estrogen level and endometrium in two groups. According to previous studies, calcitriol (1,25 (OH) 2 vitamin D) directly led to the production of estrogen and progesterone in cultured human ovary and placenta cells, which may enhance the luteinization of granulosa cells and ameliorate endometrial environment (33). The reason for the discrepancy could be there are few studies on the relationship about vitamin D and sex hormones, and more studies are still required. In addition, the difference in the basic treatment and monitoring days of each trail trial were included in this study, after further analyzing, the heterogeneity eliminated and the endometrial thickness of vitamin D supplementation group was increased compared with the control group. Only one study suggested that progesterone increased after vitamin D use, which was consistent with previous reports. Besides, the increase of progesterone may reduce the abortion rate.

There was no difference in the level of AMH between the vitamin D intervention group and the control group in present study. Some studies show that serum vitamin D is positively correlated with AMH, while in VD-deficient women with PCOS, VD supplementation improves the abnormally elevated AMH (44, 45). There is discrepancy in our study and previous studies. The reason may be that patients’ conditions possess their own characteristics, and the population analyzed in previous studies has no fertility requirements. Besides, age may be one reason for inconsistency.

In present study, the LH and FSH in the vitamin D intervention group were notably lower than those in the control group in PCOS patients. For extraordinary heterogeneity of LH, we executed sensitivity analysis. After removing Liu shengxian’s study, the heterogeneity decreased to 0%. The heterogeneousness may come from the elementary treatment whether use short-acting contraceptives and ovulation induction drugs or other medicine that affect sex hormones. It indicates that vitamin D supplementation may reduce LH level based on using short-acting contraceptives or ovulation induction drugs. Departed studies indicate that serum vitamin D level is negatively correlated with serum LH and FSH, but no statistical difference (46). While other findings demonstrate that LH and FSH levels are not related to vitamin D level (47), that may be due to most of the previous studies are women of healthy childbearing age, while our study focus on PCOS patients. The possible mechanism involved in vitamin D ameliorating LH of PCOS is that vitamin D attenuates the adverse effects of AGEs on human granulosa cell (GCs) steroids by down-regulating the expression of pro-inflammatory cell membrane receptor for AGEs (RAGE) (48). In addition, in human cumulus granulosa cells, vitamin D increases aromatase activity (49), while the expression of AMH receptor and FSH receptor decrease, thus promoting androgen converts to estrogen. Besides, vitamin D offsets the inhibition of AMH on GC differentiation and allows follicles to maturate and ovulate (49).

Though our study is the first systematic evaluation and meta-analysis of vitamin D on pregnancy in PCOS patients, some limitations should be treated carefully. Firstly, the quality of included studies could be higher. Second, the data of some indicators such as the cumulative pregnancy rate was not published, which induces the analysis limited.

## 5 Conclusion

In conclusion, the current evidence demonstrated that vitamin D supplementation may improve the ovulation and pregnancy rate of PCOS patients on the basis of routine treatment, providing a certain basis for clinical use of vitamin D in PCOS patients in the future. However, due to the quality and heterogeneity of the included studies, more valuable studies are still needed.

## Data availability statement

The original contributions presented in the study are included in the article/**Supplementary Material**. Further inquiries can be directed to the corresponding authors.

## Author contributions

MY and JP contributed to the selection of articles, evaluation of evidence quality, and extraction of data. MY and XS performed the data analyses and wrote the manuscript. JZ contributed to the conception of the study. SZ modified this article. DL, LX, and JZ helped perform the analyses with constructive discussions. All authors contributed to the article and approved the submitted version.
