# Supplementary material for: Effects of vitamin D supplementation on ovulation and pregnancy in women with polycystic ovary syndrome: a systematic review and meta-analysis
Source: Front Endocrinol (Lausanne). 2023 Aug 1;14:1148556. doi: 10.3389/fendo.2023.1148556 (PMC10430882; doi:10.3389/fendo.2023.1148556)
Supplement: Supplementary file 1 [file DataSheet_1.doc]

**Supplementary Information**

**Search strategy**

PubMed：

**((Polycystic Ovary Syndrome OR Ovary Syndrome, Polycystic OR Syndrome, Polycystic Ovary OR Polycystic ovary disease OR Stein-Leventhal Syndrome OR Stein Leventhal Syndrome OR Syndrome, Stein-Leventhal OR Sclerocystic Ovarian Degeneration OR Ovarian Degeneration, Sclerocystic OR Sclerocystic Ovary Syndrome OR Polycystic Ovarian Syndrome OR Ovarian Syndrome, Polycystic OR Polycystic Ovary Syndrome 1 OR Sclerocystic Ovaries OR Ovary, Sclerocystic OR Sclerocystic Ovary OR hyperandrogenism OR Hypertrichosis OR Hirsutism OR "PCOS" OR "PCO") AND (("vitamin d"[MeSH Terms] OR "vitamin d"[All Fields] OR "cholecalciferol"[All Fields]))) AND ((Randomized Controlled Trial[ptyp] AND "humans"[MeSH Terms])**

**Ovid （embase and Cochrane）**

1. exp Polycystic Ovary Syndrome/
2. Polycystic Ovar$.tw.
3. (pco or pcos).tw.
4. (sclerocystic adj3 ovar$).tw.
5. stein leventhal.tw.
6. 1 or 2 or 3 or 4 or 5
7. exp vitamin D/
8. exp colecalciferol/
9. cholecalciferol.mp. [mp=title, original title, abstract, mesh headings, heading words, keyword]
10. 7 or 8 or 9
11. 6 and 10
12. randomized controlled trial/ or (trial* or random* or RCT*).mp.
13. (double$ adj4 blind$).mp.
14. (triple$ adj4 blind$).mp.
15. 12 or 13 or 14
16. 11 and 15

**Outcome**

1. Pregnancy related outcomes

1. Chemical pregnancy rate

Biochemical pregnancy rate was reported in only 2 studies, with 81 subjects in the intervention group and 78 participants in the control group. Random effects model meta-analysis showed there were no significant difference between vitamin D supplemented and control groups in chemical pregnancy rate [RR = 0.95, 95% CI (0.55, 1.63), I² = 86%] (Supplementary table 1).

1. Incidence of pregnancy complications

A total of three studies reported the incidence of gestational hypertension and showed that there was no significant difference in PCOS patients treated with vitamin D compared with controls [RR = 0.40, 95% CI (0.15, 1.11), P = 0.08, I² = 0%].

Three studies revealed the incidence of gestational diabetes mellitus (GDM) and demonstrated that the incidence of GDM was not significantly changed in the intervention group compared with controls [RR = 0.27, 95% CI (0.05, 1.39), P = 0.11, I² = 0%] (Supplementary table 1).

1. The early miscarriage rate

Five RCTs exposed on the rate of early miscarriage, including 172 participants in the intervention group and 135 in the control group. The meta-analysis with a fixed effects model elucidated that the early miscarriage rate was notably lower in the vitamin D supplemented group compared with the control group [RR = 0.44, 95% CI (0.30, 0.66), P < 0.0001, I² = 0%] (Supplementary table 1). Only Ruili Chen ^[11]^ mentioned the baseline of Vitamin D deficiency. Subgroup analyses showed that, daily vitamin D ≤ 2000 IU, [RR = 0.34, 95% CI (0.19, 0.59), P = 0.0001], and only Rasheedy’s study^[10]^ with daily vitamin D > 2000 IU, [RR = 0.65, 95% CI (0.37, 1.14), P = 0.13]; P = 0.001], 8 weeks^[12, 13]^ [RR = 0.31, 95% CI (0.15, 0.63), P = 0.001], 12 weeks^[11, 14]^ [RR = 0.38, 95% CI (0.16, 0.94), P = 0.04], and only one^[10]^ used vitamin D greater than 12 weeks, [RR = 0.65, 95% CI (0.37, 1.14), P = 0.13] (Supplementary table 1).

1. Fertilization rate

Fertilization rate was displayed in three studies, of which 1267 cycles in the intervention group and 1204 cycles in the control group were included. The fixed effects model demonstrated no significant difference in fertilization rates between the two groups [RR=1.05, 95%CI (1.00, 1.10)，p=0.04，I²=0%] (Supplementary table 1).

1. The cleavage rate

The cleavage rate was showed in 2 studies, in which 582 cycles in the vitamin D group and 526 cycles in the control group, fixed effects model unveiled no significant change in two groups [RR=1.03, 95%CI (0.99, 1.06)，I²=42%] (Supplementary table 1).

1. High quality embryo

The rate of high quality embryos was reported in 2 RCTs, which included 943 cycles in the intervention group and 840 cycles in the control group. The results of the fixed effects model elucidated that there was no significant difference with or without vitamin D supplementation group compared with the control group [RR=1.08, 95%CI (0.98, 1.20)，p=0.10，I²=0%] (Supplementary table 1).

1. Preterm birth rate

Only 2 studies appeared the preterm birth rate, and the results suggested a obviously reduction in the vitamin D intervention group [RR=0.38, 95%CI (0.21, 0.70)，p=0.002，I²=0%] (Supplementary table 1).

2.Ovulation related outcomes

(1) Matured oocytes rate

A total of 7 RCTs reported the matured oocytes rate, including 2955 in the intervention group and 2952 in the control group. Fixed effect model showed that the oocyte maturation rate of PCOS patients was ameliorated [RR=1.08 ,95%CI (1.03, 1.13)，p=0.002，I²=27%] (Supplementary table 1).

1. The endometrial thickness

Altogether, there were 7 RCTs mentioned the endometrial thickness, involving 392 subjects in the vitamin D group and 387 in control group (Supplementary table 1). The random effect model showed that there was no significant difference of endometrial thickness in two groups [MD=0.77 ,95%CI (-0.23, 1.77)，p=0.13，I²=96%]. For high heterogeneity existed, we performed subgroup analyses based on whether took ovulation induction drugs and the period of ultrasonography in the menstrual cycle. The result revealed heterogeneity was still remarkable, and no association was found in subgroups under the random effect model. However, when combined two factors, that is, when examined at D10 while letrozole and HMG was used, the I² dropped to 0%. Under the fixed effect model, the endometrial thickness in the vitamin D supplement group were thicker than that in the control group [MD=2.70 ,95%CI (2.35, 3.05)，p＜0.00001]. Thus, it suggests that the specific protocol for ovulation induction and the period of examination are both factors affecting the heterogeneity of endometrial thickness (Supplementary table 1).

3. Glycometabolism related outcomes

1. Fasting blood glucose (FBG)

We found there was no significant difference in fasting blood glucose levels between the vitamin D supplement group and the control group by pooling the available 3 studies, with 146 cases in the test group and 146 cases in the control group [MD= -0.52, 95%CI (-1.38, 0.34) ，p=0.23，I²=91%] (Supplementary table 1).

1. Fasting blood insulin (FINS)

Three studies showed fasting insulin levels, consisted of 146 cases in the vitamin D group and 146 cases in the control group. The random effect model showed no apparent difference between two groups [MD= -0.02 ,95%CI(-0.50, 0.46)，p=0.93，I²=72%] (Supplementary table 1).

(3) Homeostasis model assessment as an index of insulin resistance (HOMA-IR)

We collected the only 2 researches reported HOMA-IR, involving 75 participants in the intervention group and 76 in the control group. The random effect demonstrated no significant difference in HOMA-IR between two groups [MD= -0.23 ,95%CI(-1.77, 1.32)，p=0.77，I²=73%] (Supplementary table 1).

Supplementary table 1. Vitamin D supplement compared with control for PCOS patients

| Outcome | | Study included number | Heterogeneity | | Effect model | Meta analysis | |
| --- | --- | --- | --- | --- | --- | --- | --- |
|  |  |  | P value | I^2^ |  | Relative effect (95%CI) | P value |
| Pregnancy related outcomes | Chemical pregnancy rate | 2 | 0.007 | 86% | Random | 0.95 (0.55, 1.63) | 0.84 |
|  | Premature miscarriage rate | 5 | 0.53 | 0% | Fixed | 0.44 (0.30, 0.66) | ＜0.0001 |
|  | Premature delivery rate | 2 | 0.89 | 0% | Fixed | 0.38 (0.21, 0.70) | 0.002 |
| Incidence of pregnancy complications | Gestational hypertension | 3 | 0.81 | 0% | Fixed | 0.40 (0.15, 1.11) | 0.08 |
|  | Gestational diabetes mellitus | 2 | 0.54 | 0% | Fixed | 0.27 (0.05, 1.39) | 0.11 |
| IVF related outcomes | Fertilization rate | 3 | 0.87 | 0% | Fixed | 1.05 (1.00, 1.10) | 0.04 |
|  | Cleavage rate | 2 | 0.19 | 42% | Fixed | 1.03 (0.99, 1.06) | 0.17 |
|  | High quality embryo rate | 3 | 0.76 | 0% | Fixed | 1.08 (0.98, 1.20) | 0.10 |
| Ovulation related outcomes | Matured oocytes rate | 7 | 0.23 | 27% | Fixed | 1.08 (1.03, 1.13) | 0.002 |
| Sex hormone related outcomes | Endometrial thickness | 7 | ＜0.00001 | 96% | Random | 0.77(-0.23, 1.77) | 0.13 |
| Glycometabolism  related outcomes | FBG | 3 | ＜0.0001 | 91% | Random | -0.52(-1.38, 0.34) | 0.23 |
|  | FINS | 3 | 0.03 | 72% | Random | -0.02(-0.50, 0.46) | 0.93 |
|  | HOMA-IR | 2 | 0.05 | 73% | Random | -0.23 (-1.77, 1.32) | 0.77 |

FBG: fasting blood glucose, FINS: fasting blood insulin, HOMA-IR: homeostasis model assessment as an index of insulin resistance.
